# Prediction of treatment responses to neoadjuvant chemotherapy in triple-negative breast cancer by analysis of immune checkpoint protein expression

**DOI:** 10.1186/s12967-018-1458-y

**Published:** 2018-04-04

**Authors:** Yuka Asano, Shinichiro Kashiwagi, Wataru Goto, Koji Takada, Katsuyuki Takahashi, Tamami Morisaki, Hisakazu Fujita, Tsutomu Takashima, Shuhei Tomita, Masahiko Ohsawa, Kosei Hirakawa, Masaichi Ohira

**Affiliations:** 1 Department of Surgical Oncology, Osaka City University Graduate School of Medicine, 1-4-3 Asahi-machi, Abeno-ku, Osaka, 545-8585 Japan; 2 Department of Pharmacology, Osaka City University Graduate School of Medicine, 1-4-3 Asahi-machi, Abeno-ku, Osaka, 545-8585 Japan; 3 Department of Scientific and Linguistic Fundamentals of Nursing, Osaka City University Graduate School of Nursing, 1-5-17 Asahi-machi, Abeno-ku, Osaka, 545-0051 Japan; 4 Department of Diagnostic Pathology, Osaka City University Graduate School of Medicine, 1-4-3 Asahi-machi, Abeno-ku, Osaka, 545-8585 Japan

**Keywords:** Immune checkpoint, Triple-negative breast cancer, Pathological complete response, Neoadjuvant chemotherapy, PD-1, PD-L1

## Abstract

**Background:**

“Avoiding immune destruction” has recently been established as one of the hallmarks of cancer. The programmed cell death (PD)-1/programmed cell death-ligand (PD-L) 1 pathway is an important immunosuppression mechanism that allows cancer cells to escape host immunity. The present study investigated how the expressions of these immune checkpoint proteins affected responses to neo-adjuvant chemotherapy (NAC) in breast cancer.

**Methods:**

A total of 177 patients with resectable early-stage breast cancer were treated with NAC. Estrogen receptor, progesteron receptor, human epidermal growth factor receptor 2, Ki67, PD-L1, PDL-2 and PD-1 status were assessed by immunohistochemistry.

**Results:**

There were 37 (20.9%) patients with high PD-1 expression, 42 (23.7%) patients had high PD-L1 expression, and 52 (29.4%) patients had high PD-L2 expression. The patients with high PD-1 and PD-L1 expressions had a significantly higher rate of triple-negative breast cancer (TNBC) (p = 0.041) (p < 0.001). In TNBC, patients with high PD-1 and PD-L1 expressions had significantly higher rates of non-pCR (p = 0.003) (p < 0.001). Univariate analysis showed that PD-1 and PD-L1 expressions also significantly shortened disease free survival in TNBC (p = 0.048, HR = 3.318) (p = 0.007, HR = 8.375). However, multivariate analysis found that only PD-L1 expression was an independent prognostic factor (p = 0.041, HR = 9.479).

**Conclusions:**

PD-1 and PD-L1 expressions may be useful as biomarkers to predict treatment responses to NAC in breast cancer. Above all, PD-L1 expression may also be useful as biomarkers for more effective chemotherapy in TNBC.

**Electronic supplementary material:**

The online version of this article (10.1186/s12967-018-1458-y) contains supplementary material, which is available to authorized users.

## Background

Various immunosuppressive factors from cancer cells in the tumor microenvironment inhibit host immune responses to cancer [1, 2]. Several immune checkpoints exist in immune response pathways, and negative costimulatory molecules such as cytotoxic T-lymphocyte-associated protein (CTLA)-4 and PD-1 are important checkpoints in limiting self-immune responses [3, 4]. Immunotherapy is effective not only in malignant melanoma and renal cell carcinoma (RCC), but anti-tumor effects have also been demonstrated in a variety of other cancers. Immune checkpoint inhibitor therapy, which turns anti-tumor T cells into effectors, has completely changed the role of cancer immunotherapy in clinical practice [5–8].

Breast cancer was not previously regarded as a tumor associated with abnormal immunity [9]. However, in a phase I trial in TNBC, the PD-1 inhibitor pembrolizumab showed anti-tumor activity, and correlations between PD-1 and PD-L1 (B7-H1) expressions and outcomes were reported [10–13]. Thus, immune checkpoint inhibitor therapy is expected to play a major role in the tailored treatment of breast cancer.

Immunohistological analysis has shown that PD-L1 expression is induced in most solid tumors, including malignant melanoma, ovarian cancer, lung cancer, RCC, and breast cancer. PD-L1 expression in cancer cells has been correlated with cancer progression, the occurrence of metastases, and survival rates [14–17]. In addition, tumor-infiltrating lymphocytes (TILs) include PD-1-positive lymphocytes, and a correlation between them and prognosis in breast cancer has been reported [11].

The effect of the tumor immune environment not only on immunotherapy effectiveness, but also on conventional anti-tumor therapy effectiveness and prognosis, has recently been demonstrated [18]. Thus, improvement of the tumor immune environment is important. In other words, the tumor immune environment plays a role in the anti-tumor effects of conventional anti-tumor drugs. Moreover, immune checkpoint proteins such as PD-1, PD-L1, and PD-L2 (B7-H2) may play an important role in improving the tumor immune environment. Given this background, the clinical significance of immune checkpoint protein expression was investigated in patients receiving NAC for breast cancer using conventional anti-cancer drugs, and whether this would be useful as a marker to predict treatment response was evaluated.

## Methods

### Patient background

A total of 177 patients with resectable, early-stage breast cancer diagnosed as stage IIA (T1, N1, M0 or T2, N0, M0), IIB (T2, N1, M0 or T3, N0, M0), or IIIA (T1-2, N2, M0 or T3, N1-2, M0) were treated with NAC between 2007 and 2013. Tumor stage and T and N factors were stratified based on the TNM Classification of Malignant Tumors, UICC Sixth Edition [19]. Breast cancer was confirmed histologically by core needle biopsy and staged by systemic imaging studies using computed tomography (CT), ultrasonography (US), and bone scintigraphy. Breast cancer was classified into subtypes according to the immunohistochemical expression of ER, PgR, HER2, and Ki67. Based on their immunohistochemical expression, the tumours are categorized into the immunophenotypes luminal A (ER+ and/or PgR+, HER2−, Ki67-low), luminal B (ER+ and/or PgR+, HER2+) (ER+ and/or PgR+, HER2−, Ki67-high), HER2-enriched (ER−, PgR−, and HER2+), and TNBC (negative for ER, PgR and HER2) [20]. In this study, HER2-enriched and luminal B (ER+ and/or PgR+, HER2+) were considered as HER2-positive breast cancer (HER2^+^BC).

All patients received a standardized protocol of NAC consisting of four courses of FEC100 (500 mg/m^2^ fluorouracil, 100 mg/m^2^ epirubicin, and 500 mg/m^2^ cyclophosphamide) every 3 weeks, followed by 12 courses of 80 mg/m^2^ paclitaxel administered weekly [21, 22]. Forty-five patients had HER2^+^BC and were additionally administered weekly (2 mg/kg) or tri-weekly (6 mg/kg) trastuzumab during paclitaxel treatment [23]. All patients underwent chemotherapy as outpatients. Therapeutic anti-tumor effects were assessed according to the response evaluation criteria in solid tumors (RECIST) criteria [24]. Pathological complete response (pCR) was defined as the complete disappearance of the invasive compartment of the lesion with or without intraductal components, including in the lymph nodes. Patients underwent mastectomy or breast-conserving surgery after NAC. All patients who underwent breast-conserving surgery were administered postoperative radiotherapy to the remnant breast. Overall survival (OS) time was the period from the initiation of NAC to the time of death from any cause. Disease-free survival (DFS) was defined as freedom from all local, loco-regional, and distant recurrences. All patients were followed up by physical examination every 3 months, US every 6 months, and CT and bone scintigraphy annually. The median follow-up period for the assessment of OS was 3.4 years (range 0.6–6.0 years), and for DFS it was 3.1 years (range 0.1–6.0 years). This study has been conducted in our institution, Osaka City University Graduate School of Medicine, Osaka, Japan, according to the reporting recommendations for tumor marker prognostic studies (REMARK) guidelines and a retrospectively written research, pathological evaluation, and statistical plan [25].

### Immunohistochemistry

All patients underwent a core needle biopsy prior to NAC, and they underwent curative surgery involving a mastectomy or conservative surgery with axillary lymph node dissection after NAC at the Osaka City University. Immunohistochemical studies were performed as previously described on core needle biopsy specimens [26]. Tumour specimens were fixed in 10% formaldehyde solution and embedded in paraffin (FFPE), and 4-µm-thick sections were mounted onto glass slides. Slides were deparaffinized in xylene and heated for 20 min (105 °C, 0.4 kg/m^2^) in an autoclave in Target Retrieval Solution (Dako, Carpinteria, CA, USA). Specimens were then incubated with 3% hydrogen peroxide in methanol for 15 min to block the endogenous peroxidase activity, then incubated in 10% normal goat or rabbit serum to block non-specific reactions.

Primary monoclonal antibodies directed against ER (clone 1D5, dilution 1:80; Dako, Cambridge, UK), PgR (clone PgR636, dilution 1:100; Dako), HER2 (HercepTest™; Dako), Ki67 (clone MIB-1, dilution 1:00; Dako), PD-1 (clone NAT105, dilution 1:100; Abcam, Cambridge, UK), PD-L1 (clone 27A2, dilution 1:100; MBL, Nagoya, Japan), and PD-L2 (clone #176611, dilution 1:100; R&D Systems, Minneapolis, MN) were used. Tissue sections were incubated with each antibody for 70 min at room temperature or overnight at 4 °C, then incubated with horseradish peroxidase-conjugated anti-rabbit or anti-mouse Ig secondary antibodies (HISTOFINE (PO)™ kit; Nichirei, Tokyo, Japan). Slides were subsequently treated with streptavidin-peroxidase reagent and incubated in phosphate-buffered saline-diaminobenzidine and 1% hydrogen peroxide (v/v), followed by counterstaining with Mayer’s haematoxylin. Positive and negative controls were carried out on FFPE lymph node tissues using corresponding monoclonal antibody and mouse isotype IgG.

### Immunohistochemical scoring

Immunohistochemical scoring was performed by two pathologists specialized in mammary gland pathology, using the blind method to confirm the objectivity and reproducibility of diagnosis. The cut-off value for ER and PgR positivity was set at ≥ 1% in accordance with previous studies [27]. HER2 expression was scored according to the accepted grading system (0, no reactivity, or membranous reactivity in less than 10% of cells; 1 +, faint/barely perceptible membranous reactivity in ≥ 10% of cells or reactivity in only part of the cell membrane; 2 +, weak to moderate complete or basolateral membranous reactivity in ≥ 10% of tumour cells; or 3 +, strong complete or basolateral membranous reactivity in ≥ 10% of tumour cells). HER2 expression was considered positive if the immunostaining score was 3 +, or in cases where the score was 2 + and included gene amplification via fluorescent in situ hybridization (FISH). For FISH analyses, each copy of the *HER2* gene and its centromere 17 (CEP17) reference were counted. The interpretation followed the criteria of the ASCO/CAP guidelines for HER2 IHC classification for breast cancer: positive if the HER2/CEP17 ratio was higher than 2.0 [28]. A Ki67-labeling index ≥ 14% was classified as positive [20].

**Fig. 1:**
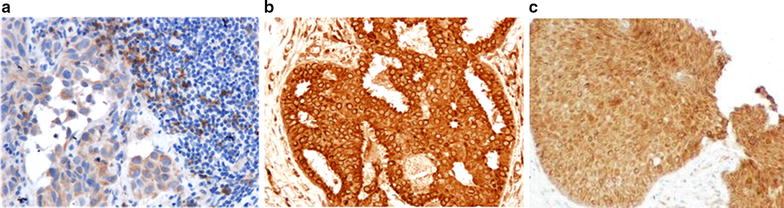
To evaluate PD-1, PD-L1 and PD-L2 expression. These pictures were judged to be positive for expression (400-times magnification). Immunohistochemical staining using each monoclonal antibodies: **a** PD-1, **b** PD-L1, **c** PD-L2

Histopathologic analysis of TILs was evaluated on a single full-face hematoxylin and eosin (HE)-stained tumor section using criteria described [29–31]. To evaluate PD-1 expression, five stained areas were selected, and the number of TILs in stroma surrounding the stained cancer cells was quantitatively measured in each field under 400-times magnification (Fig. 1a). The median value of the average each field was determined, and that number was set as a cutoff value. To evaluate PD-L1 and PD-L2 expression, 3 fields of view (FOVs) in darkly stained areas were selected, and the percentage of cancer cells stained with anti-PD-L1 antibody and anti-PD-L2 antibody in each FOVs was measured under 400-times magnified microscopy (Fig. 1b, c). Based on previous reports, ≥ 10% was defined as high expression, and < 10% was defined as low expression [12, 32].

### Statistical analysis

Statistical analysis was performed using the SPSS version 19.0 statistical software package (IBM, Armonk, NY). The associations between PD-1, PD-L1 and PD-L2 and clinicopathological variables were evaluated using χ^2^ tests. Multivariate analysis of pCR was carried out using a binary logistic regression model. The Kaplan–Meier method was used to estimate DFS and OS, and the results were compared between groups using log-rank tests. Multivariate analysis of prognostic factors was carried out using a Cox regression model. A *p* value < 0.05 was considered significant. Cutoff values for different biomarkers included in this study were chosen before the statistical analysis.

## Results

### Clinicopathological responses of primary breast cancers to NAC

The subtype in 177 patients who received NAC was TNBC in 61 (34.5%) patients and HER2^+^BC in 45 (25.4%) patients. Regarding treatment response, 67 (37.9%) patients had a pCR, and 110 (62.1%) patients had a non-pCR. According to subtype, 28 (45.9%) TNBC patients and 18 (40.0%) HER2^+^BC patients had a pCR.

### Immune checkpoint protein expression in all breast cancers

TIL PD-1 expression ranged from 0 to 68. The median value of the average in 3 FOVs was 6. There were 37 (20.9%) patients with high PD-1 expression (≥ 6) and 140 (79.1%) patients with low PD-1 expression (< 6). In addition, 42 (23.7%) patients had high PD-L1 expression, and 52 (29.4%) patients had high PD-L2 expression.

Evaluation based on clinicopathologic features showed that patients with high PD-1 and PD-L1 expressions had a significantly higher rate of TNBC (p = 0.041) (p < 0.001) and HER2^+^BC (p = 0.004) (p = 0.004). Patients with PD-L1 expression had a significantly higher rate of non-pCR (p < 0.001), and PD-1 and PD-L2 expressions were greater (p < 0.001) (p < 0.001) (Table 1). There were no significant differences for other clinicopathologic features.

**Table 1:** Correlation between clinicopathological features and PD-1, PD-L1, and PD-L2 expression in 177 all breast cancers

Parameters	PD-1		*p* value	PD-L1		*p* value	PD-L2		*p* value
	Positive (*n* = 37)	Negative (*n* = 140)		Positive (*n* = 42)	Negative (*n *= 135)		Positive (*n* = 53)	Negative (*n *= 124)	
Intrinsic subtype									
TNBC	18 (48.6%)	43 (30.7%)	0.041	24 (57.1%)	37 (27.4%)	< 0.001	20 (37.7%)	41 (33.1%)	0.549
Non-TNBC	19 (51.4%)	97 (69.3%)		18 (42.9%)	98 (72.6%)		33 (62.3%)	83 (66.9%)	
Intrinsic subtype									
HER2^+^BC	3 (81.1%)	42 (30.0%)	0.004	4 (9.5%)	41 (30.4%)	0.004	16 (30.2%)	29 (23.4%)	0.341
Non-HER2^+^ BC	34 (18.9%)	98 (70.0%)		38 (90.5%)	94 (69.6%)		37 (69.8%)	95 (76.6%)	
Age at operation									
≤ 56	15 (40.5%)	72 (51.4%)	0.239	20 (47.6%)	67 (49.6%)	0.820	28 (52.8%)	59 (47.6%)	0.522
> 56	22 (59.5%)	68 (48.6%)		22 (52.4%)	68 (50.4%)		25 (47.2%)	65 (52.4%)	
Menopause									
Negative	14 (37.8%)	58 (41.4%)	0.693	18 (42.9%)	54 (40.0%)	0.742	23 (43.4%)	49 (39.5%)	0.630
Positive	23 (62.2%)	82 (58.6%)		24 (57.1%)	81 (60.0%)		30 (56.6%)	75 (60.5%)	
Tumor size (cm)									
≤ 2	5 (13.5%)	19 (13.6%)	0.993	6 (14.3%)	18 (13.3%)	0.875	9 (17.0%)	15 (12.1%)	0.385
> 2	32 (86.5%)	121 (86.4%)		36 (85.7%)	117 (86.7%)		44 (83.0%)	109 (87.9%)	
Lymph node status									
Negative	7 (18.9%)	34 (24.3%)	0.491	10 (23.8%)	31 (23.0%)	0.910	12 (22.6%)	29 (23.4%)	0.914
Positive	30 (81.1%)	106 (75.7%)		32 (76.2%)	104 (77.0%)		41 (77.4%)	95 (76.6%)	
Nuclear grade									
1, 2	26 (70.3%)	111 (79.3%)	0.244	31 (73.8%)	106 (78.5%)	0.524	39 (73.6%)	98 (79.0%)	0.427
3	11 (29.7%)	29 (20.7%)		11 (26.2%)	29 (21.5%)		14 (26.4%)	26 (21.0%)	
Ki67									
≤ 14%	13 (35.1%)	61 (43.6%)	0.355	16 (38.1%)	58 (43.0%)	0.576	18 (34.0%)	56 (45.2%)	0.167
> 14%	24 (64.9%)	79 (56.4%)		26 (61.9%)	77 (57.0%)		35 (66.0%)	68 (54.8%)	
Pathological response									
pCR	9 (24.3%)	58 (41.4%)	0.056	6 (14.3%)	61 (45.2%)	< 0.001	24 (45.3%)	43 (34.7%)	0.183
Non-pCR	28 (75.7%)	82 (58.6%)		36 (85.7%)	74 (54.8%)		29 (54.7%)	81 (65.3%)	
PD-1									
Negative	Not determined	Not determined		13 (31.0%)	127 (94.1%)	< 0.001	31 (58.5%)	109 (87.9%)	< 0.001
Positive				29 (69.0%)	8 (5.9%)		22 (41.5%)	15 (12.1%)	
PD-L1									
Negative	8 (21.6%)	127 (90.7%)	< 0.001	Not determined	Not determined		25 (47.2%)	110 (88.7%)	< 0.001
Positive	29 (78.4%)	13 (9.3%)					28 (52.8%)	14 (11.3%)	
PD-L2									
Negative	15 (40.5%)	109 (77.9%)	< 0.001	14 (33.3%)	110 (81.5%)	< 0.001	Not determined	Not determined	
Positive	22 (59.5%)	31 (22.1%)		28 (66.7%)	25 (18.5%)				

*TNBC* triple-negative breast cancer, *HER2* human epidermal growth factor receptor 2, *BC* breast cancer, *pCR* pathological complete response, *PD-1* programmed cell death-1, *PD-L* programmed cell death-ligand

**Fig. 2:**
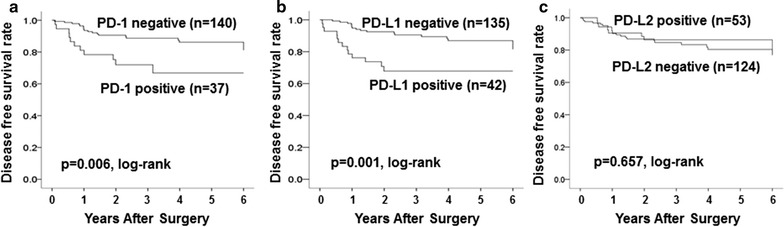
Disease-free survival (DFS) was significantly longer in patients with low, compared to high, PD-1 and PD-L1 expressions (p = 0.006, log-rank) (**a**) (p = 0.001, log-rank) (**b**). DFS did not differ significantly between patients with low vs high PD-L2 expression (p = 0.657, log-rank) (**c**)

DFS was significantly longer in patients with low, compared to high, PD-1 and PD-L1 expressions (p = 0.006, log-rank) (p = 0.001, log-rank) (Fig. 2a, b). OS was also significantly longer in patients with low, compared to high, PD-1 and PD-L1 expressions (p = 0.048, log-rank) (p = 0.022, log-rank) (Additional file 1: Fig. S1A, B). DFS and OS did not differ significantly between patients with low vs high PD-L2 expression (p = 0.657, log-rank) (p = 0.615, log-rank) (Fig. 2c) (Additional file 1: Fig. S1C).

Univariate analysis showed that PD-1 and PD-L1 expressions were associated with significantly shorter DFS (p = 0.008, HR = 2.752) (p = 0.002, HR = 3.194). However, although multivariate analysis showed that lymph node metastases were an independent poor prognostic factor (p = 0.046, HR = 4.330), PD-1 and PD-L1 expressions were not independent prognostic factors (p = 0.492, HR = 1.415) (p = 0.084, HR = 2.613) (Table 2).

**Table 2:** Univariate and multivariate analysis with respect to progression free survival in 177 all breast cancers

Parameters	Univariate analysis			Multivariate analysis		
	Hazard ratio	95% CI	*p* value	Hazard ratio	95% CI	*p* value
Subtype TNBC vs non-TNBC	1.213	0.577–2.550	0.611	0.849	0.387–1.861	0.683
Subtype HER2^+^BC vs non-HER2^+^BC	0.421	0.147–1.206	0.107	0.552	0.181–1.686	0.297
Lymph node status Positive vs negative	4.157	0.990–17.456	0.052	4.330	1.027–18.263	0.046
Pathological response pCR vs Non-pCR	0.611	0.279–1.336	0.217	0.854	0.352–2.072	0.728
PD-1 Positive vs negative	2.752	1.300–5.826	0.008	1.415	0.526–3.811	0.492
PD-L1 Positive vs negative	3.194	1.544–6.607	0.002	2.613	0.879–7.766	0.084

*CI* confidence interval, *TNBC* triple-negative breast cancer, *HER2* human epidermal growth factor receptor 2, *BC* breast cancer, *pCR* pathological complete response, *PD-1* programmed cell death-1, *PD-L* programmed cell death-ligand

### Immune checkpoint protein expression in triple-negative breast cancer

Among the 61 patients with TNBC, 18 (29.5%) had high PD-1 expression, 24 (39.3%) had high PD-L1 expression, and 20 (32.8%) had high PD-L2 expression. Analysis of clinicopathologic features showed that the high PD-1 expression group was significantly older (p = 0.016), and that patients with high PD-L1 expression had a significantly lower Ki67 index (p = 0.005). Patients with high PD-1 and PD-L1 expressions had significantly higher rates of non-pCR (p = 0.003) (p < 0.001). PD-1 expression was significantly correlated with PD-L1 expression (p < 0.001), it but was not correlated with PD-L2 expression (Table 3).

**Table 3:** Correlation between clinicopathological features and PD-1, PD-L1, and PD-L2 expression in 61 triple-negative and 45 HER2-positive breast cancers

Parameters	PD-1			PD-L1			PD-L2		
	Positive (*n* = 18)	Negative (*n* = 43)	*p* value	Positive (*n* = 24)	Negative (*n *= 37)	*p* value	Positive (*n* = 20)	Negative (*n *= 41)	*p* value
TNBC (n = 61)									
Age at operation									
≤ 56	4 (22.2%)	24 (55.8%)	0.016	10 (41.7%)	18 (48.6%)	0.593	12 (60.0%)	16 (39.0%)	0.123
> 56	14 (77.8%)	19 (44.2%)		14 (58.3%)	19 (51.4%)		8 (40.0%)	25 (61.0%)	
Menopause									
Negative	4 (22.2%)	18 (41.9%)	0.121	9 (37.5%)	13 (35.1%)	0.851	8 (40.0%)	14 (34.1%)	0.655
Positive	14 (77.8%)	25 (58.1%)		15 (62.5%)	24 (64.9%)		12 (60.0%)	27 (65.9%)	
Tumor size (cm)									
≤ 2	3 (16.7%)	4 (9.3%)	0.337	3 (12.5%)	4 (10.8%)	0.573	3 (15.0%)	4 (9.8%)	0.416
> 2	15 (83.3%)	39 (90.7%)		21 (87.5%)	33 (89.2%)		17 (85.0%)	37 (90.2%)	
Lymph node status									
Negative	3 (16.7%)	8 (18.6%)	0.586	5 (20.8%)	6 (16.2%)	0.647	3 (15.0%)	8 (19.5%)	0.481
Positive	15 (83.3%)	35 (81.4%)		19 (79.2%)	31 (83.8%)		17 (85.0%)	33 (80.5%)	
Nuclear grade									
1, 2	13 (72.2%)	31 (72.1%)	0.992	19 (79.2%)	25 (67.6%)	0.324	14 (70.0%)	30 (73.2%)	0.795
3	5 (27.8%)	12 (27.9%)		5 (20.8%)	12 (32.4%)		6 (30.0%)	11 (26.8%)	
Ki67									
≤ 14%	6 (33.3%)	12 (27.9%)	0.672	12 (50.0%)	6 (16.2%)	0.005	6 (30.0%)	12 (29.3%)	0.953
> 14%	12 (66.7%)	31 (72.1%)		12 (50.0%)	31 (83.8%)		14 (70.0%)	29 (70.7%)	
Pathological response									
pCR	3 (16.7%)	25 (58.1%)	0.003	3 (12.5%)	25 (67.6%)	< 0.001	10 (50.0%)	18 (43.9%)	0.654
Non-pCR	15 (83.3%)	18 (41.9%)		21 (87.5%)	12 (32.4%)		10 (50.0%)	23 (56.1%)	
PD-1									
Negative	Not determined	Not determined		9 (37.5%)	34 (91.9%)	< 0.001	12 (60.0%)	31 (75.6%)	0.210
Positive				15 (62.5%)	3 (8.1%)		8 (40.0%)	10 (24.4%)	
PD-L1									
Negative	3 (16.7%)	34 (48.4%)	< 0.001	Not determined	Not determined		10 (50.0%)	27 (65.9%)	0.234
Positive	15 (83.3%)	9 (51.6%)					10 (50.0%)	14 (34.1%)	
PD-L2									
Negative	10 (55.6%)	31 (79.1%)	0.210	14 (58.3%)	27 (73.0%)	0.234	Not determined	Not determined	
Positive	8 (44.4%)	12 (20.9%)		10 (41.7%)	10 (27.0%)				

Parameters	PD-1			PD-L1			PD-L2		
	Positive (*n* = 3)	Negative (*n* = 42)	*p* value	Positive (*n* = 4)	Negative (*n *= 41)	*p* value	Positive (*n* = 16)	Negative (*n *= 29)	*p* value
HER2^+^BC (n = 45)									
Age at operation									
≤ 56	0 (0.0%)	20 (47.6%)	0.162	1 (25.0%)	19 (46.3%)	0.394	6 (37.5%)	14 (48.3%)	0.486
> 56	3 (100.0%)	22 (52.4%)		3 (75.0%)	22 (53.7%)		10 (62.5%)	15 (51.7%)	
Menopause									
Negative	0 (0.0%)	18 (42.9%)	0.206	1 (25.0%)	17 (41.5%)	0.471	6 (37.5%)	12 (41.4%)	0.799
Positive	3 (100.0%)	24 (57.1%)		3 (75.0%)	24 (58.5%)		10 (62.5%)	17 (58.6%)	
Tumor size (cm)									
≤ 2	1 (33.3%)	5 (11.9%)	0.356	1 (25.0%)	5 (12.2%)	0.448	4 (25.0%)	2 (6.9%)	0.107
> 2	2 (66.7%)	37 (88.1%)		3 (75.0%)	36 (87.8%)		12 (75.0%)	27 (93.1%)	
Lymph node status									
Negative	1 (33.3%)	15 (35.7%)	0.715	2 (50.0%)	14 (34.2%)	0.448	5 (31.3%)	11 (37.9%)	0.455
Positive	2 (66.7%)	27 (64.3%)		2 (50.0%)	27 (65.8%)		11 (68.7%)	18 (62.1%)	
Nuclear grade									
1, 2	2 (66.7%)	33 (78.6%)	0.539	2 (50.0%)	33 (80.5%)	0.209	12 (75.0%)	23 (79.3%)	0.508
3	1 (33.3%)	9 (21.4%)		2 (50.0%)	8 (19.5%)		4 (25.0%)	6 (20.7%)	
Ki67									
≤ 14%	1 (33.3%)	23 (54.8%)	0.449	1 (25.0%)	23 (56.1%)	0.254	7 (43.8%)	17 (58.6%)	0.338
> 14%	2 (66.7%)	19 (45.2%)		3 (75.0%)	18 (43.9%)		9 (56.2%)	12 (41.4%)	
Pathological response									
pCR	0 (0.0%)	18 (42.9%)	0.206	1 (25.0%)	17 (41.5%)	0.471	11 (62.5%)	7 (24.1%)	0.005
Non-pCR	3 (100.0%)	24 (57.1%)		3 (75.0%)	24 (58.5%)		5 (37.5%)	22 (75.9%)	
PD-1									
Negative	Not determined	Not determined		1 (25.0%)	41 (100.0%)	< 0.001	13 (81.3%)	29 (100.0%)	0.039
Positive				3 (75.0%)	0 (0.0%)		3 (18.7%)	0 (0.0%)	
PD-L1									
Negative	0 (0.0%)	41 (97.6%)	< 0.001	Not determined	Not determined		12 (75.0%)	29 (100.0%)	0.012
Positive	3 (100.0%)	1 (2.4%)					4 (25.0%)	0 (0.0%)	
PD-L2									
Negative	0 (0.0%)	29 (69.0%)	0.039	0 (0.0%)	29 (70.7%)	0.012	Not determined	Not determined	
Positive	3 (100.0%)	13 (31.0%)		4 (100.0%)	12 (29.3%)				

*TNBC* triple-negative breast cancer, *HER2* human epidermal growth factor receptor 2, *BC* breast cancer, *pCR* pathological complete response, *PD-1* programmed cell death-1, *PD-L* programmed cell death-ligand

**Fig. 3:**
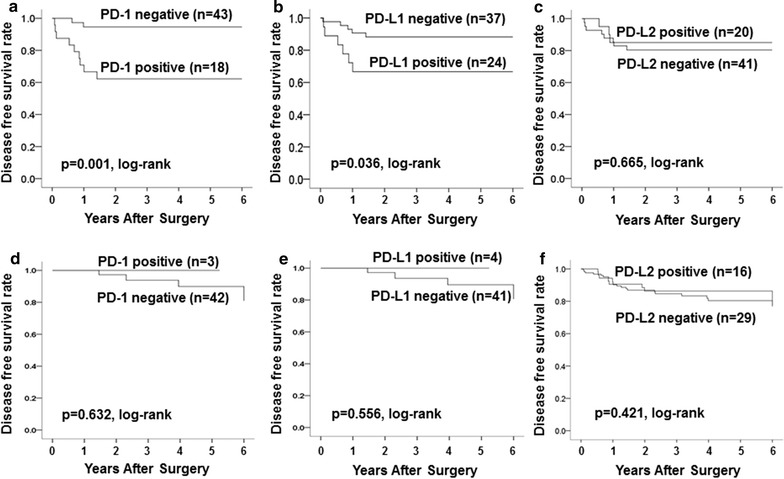
Analysis of the correlation with outcome of 61 TNBC and 45 HER2^+^BC patients. In 61 TNBC cases, DFS was significantly longer in patients with low, compared to high, PD-1 and PD-L1 expressions (p = 0.001, log-rank) (p = 0.036, log-rank) (**a**, **b**). DFS did not differ significantly between patients with low vs high PD-L2 expression (p = 0.665, log-rank) (**c**). In 45 HER2^+^BC cases, DFS was not significantly longer in patients with low, compared to patients with high, PD-1, PD-L1 and PD-L2 expressions (p = 0.632, p = 0.556, p = 0.421, log-rank, respectively) (**d**, **e**, **f**)

Analysis of outcomes showed that DFS was significantly longer in patients with low, compared to patients with high, PD-1 and PD-L1 expressions (p = 0.036, log-rank) (p = 0.001, log-rank) (Fig. 3a, b). OS was also significantly longer in patients with low, compared to patients with high, PD-1 expression (p = 0.021, log-rank), but OS was not significantly different based on PD-L1 expression (p = 0.155, log-rank) (Additional file 1: Fig. S1D, E). DFS and OS were also not significantly different based on PD-L2 expression (p = 0.665, log-rank) (p = 0.595, log-rank) (Fig. 3c) (Additional file 1: Fig. S1F).

Univariate analysis showed that PD-1 and PD-L1 expressions also significantly shortened DFS in TNBC (p = 0.048, HR = 3.318) (p = 0.007, HR = 8.375). However, multivariate analysis found that only PD-L1 expression was an independent prognostic factor (p = 0.041, HR = 9.479) (Table 4).

**Table 4:** Univariate and multivariate analysis with respect to progression free survival in 61 triple-negative and 45 HER2-positive breast cancers

Parameters	Univariate analysis			Multivariate analysis		
	Hazard ratio	95% CI	*p* value	Hazard ratio	95% CI	*p* value
TNBC (n = 61)						
Lymph node status						
Positive vs negative	0.942	0.203–4.359	0.939	1.303	0.216–7.854	0.773
Ki67						
≤ 14% vs > 14%	0.739	0.216–2.526	0.630	1.866	0.395–8.817	0.431
Pathological response						
pCR vs non-pCR	0.234	0.050–1.084	0.063	0.722	0.103–5.057	0.743
PD-1						
Positive vs negative	3.318	1.011–10.891	0.048	0.869	0.177–4.265	0.863
PD-L1						
Positive vs negative	8.375	1.807–38.812	0.007	9.479	1.092–82.320	0.041
HER2^+^BC (n = 45)						
Lymph node status						
Positive vs negative	0.603	0.318–1.145	0.122	0.641	0.318–1.294	0.215
Ki67						
≤ 14% vs > 14%	0.714	0.385–1.326	0.286	0.730	0.371–1.436	0.362
Pathological response						
pCR vs non-pCR	0.670	0.358–1.254	0.210	0.912	0.428–1.944	0.811
PD-1						
Positive vs negative	1.990	0.585–6.766	0.271	1.333	0.114–15.527	0.819
PD-L1						
Positive vs negative	1.934	0.651–5.738	0.235	1.568	0.187–13.175	0.679

*TNBC* triple-negative breast cancer, *HER2* human epidermal growth factor receptor 2, *BC* breast cancer, *CI* confidence interval, *pCR* pathological complete response, *PD-1* programmed cell death-1, *PD-L* programmed cell death-ligand

#### Immune checkpoint protein expression in HER2-positive breast cancer

Among the 45 patients with HER2^+^BC, 3 (6.7%) had high PD-1 expression, 4 (8.9%) had high PD-L1 expression, and 16 (35.6%) had high PD-L2 expression. Analysis of clinicopathologic features showed that the patients with high PD-L2 expressions had significantly higher rates of pCR (p = 0.005). PD-1 expression was significantly correlated with PD-L1 expression (p < 0.001) and PD-L2 expression (p = 0.039). And, PD-L1 expression was significantly correlated with PD-L2 expression (p = 0.012) (Table 3).

Analysis of outcomes showed that DFS was not significantly longer in patients with low, compared to patients with high, PD-1, PD-L1 and PD-L2 expressions (p = 0.632, p = 0.556, p = 0.421, log-rank, respectively) (Fig. 3d–f). OS was also not significantly longer in patients with low, compared to patients with high, PD-1, PD-L1 and PD-L2 expressions (p = 0.673, p = 0.620, p = 0.749, log-rank, respectively) (Additional file 1: Fig. S1G–I). Univariate and multivariate analysis, no factors contributing to DFS were observed (Table 4).

## Discussion

Stephen Paget in 1889 proposed the “seed and soil” theory with regard to cancer metastases, and since that time, the importance of the tumor microenvironment in cancer cell proliferation has been increasingly recognized [33]. Tumor tissue is composed not only of cancer cells, but also inflammatory cells, immunocytes, vascular and lymphatic cells, fibroblasts, and fibrous tissue, and these comprise the characteristic tumor microenvironment. The tumor immune environment affects not only the effectiveness of immunotherapy, but also the prognosis and response to other treatments, such as conventional anti-tumor drugs [18]. Thus, control and improvement of the tumor immune microenvironment are important. In other words, assessment of the tumor immune environment in each individual patient can be useful in predicting treatment responses to conventional anti-cancer drugs. Therefore, the present study investigated the immune microenvironment in breast cancer patients’ tumor tissues before receiving NAC and examined the correlation with treatment responses.

“Avoiding immune destruction” has recently been established as one of the hallmarks of cancer [33]. Cancer is controlled by immunological surveillance mechanisms at the stage of cancer cell growth and by immune responses to tumor antigens in actual cancer tissue [2]. In response to these immune responses, cancer cells themselves can alter their immunogenicity and induce immunosuppression mechanisms in the tumor microenvironment, thus enabling cancer cells to cleverly escape the host immune system, survive, and grow [1, 34].

The PD-1/PD-L1 pathway is also an important immunosuppression mechanism that allows cancer cells to escape host immunity. Because of excessive PD-1 and PD-L1 levels in the tumor microenvironment, antibody inhibition of PD-1 and PD-L1 pathways is promising for effectively reversing this immunosuppression in the tumor microenvironment [32]. Suppression of T cell activation by PD-1 signals is promoted in association with the interaction of PD-1 and its ligands PD-L1 and PD-L2 [4, 35, 36]. The present study found statistical correlations among PD-1, PD-L1, and PD-L2 expressions in all breast cancers. There might be an interaction between PD-1 and its ligand PD-L1 and PD-L2.

The present study also investigated how the expressions of these immune checkpoint proteins affected responses to NAC in breast cancer. Previous studies have shown that high, compared to low, PD-1 expression is associated with a poorer prognosis in malignant melanoma, RCC, and breast cancer [10, 11, 37]. Moreover, more reports about PD-L1 than about PD-1 suggest a correlation between PD-L1 expression and the degree of cancer malignancy and a poorer prognosis [12, 14–16]. In TNBC, PD-1 and PD-L1 expression has been reported frequently in TNBC [1, 12]. In the present study, patients with high PD-1 and PD-L1 expressions had significantly higher rates of TNBC. In addition, patients with low PD-1 and PD-L1 expressions had a significantly longer DFS. In particular, low PD-1 and PD-L1 expressions in TNBC were associated with a higher pCR rate and significantly longer DFS, and low PD-L1 expression was an independent prognostic factor. These results suggest that immune escape mediated by immune checkpoints may play a role in the biological malignancy of TNBC. Among patients who received NAC, a longer DFS in patients with low PD-L1 expression suggests increased chemotherapy sensitivity. However, as limitation of this study, it is thought that pCR which has an influence on the prognosis of TNBC after NAC was included as a factor [38, 39]. On the other hand, although HER2^+^BC showed correlation with PD-1 and PD-L1 expression, there was no effect on prognosis.

Many anti-cancer drugs have immunosuppressive effects and are not compatible with immunotherapy, but depending on their mode of administration, immunological enhancement or reversal of immunosuppression is possible [40, 41]. In the present study, a standard regimen (FEC followed by paclitaxel ± trastuzumab) was used as NAC in breast cancer. To improve immune escape on the cancer cell side, decreased sensitivity to cytotoxic T lymphocytes (CTLs) can be improved by drugs such as 5FU and paclitaxel, and so-called immunogenic cell death (ICD) of cancer cells can be induced by alkylating agents such as cyclophosphamide and anthracycline drugs [40, 42]. Moreover, to improve immune escape on the host side, paclitaxel inhibition of regulatory T cells (Tregs) and 5FU inhibition of myeloid-derived suppressor cells (MDSCs) have been reported [40, 42]. Such regimens are thought to enhance immune responses by these mechanisms. Furthermore, improvement of immune escape on the cancer cell side by PD-L1 inhibition and on the host side by PD-1 inhibition can enhance the anti-tumor effects of anti-cancer drugs.

## Conclusions

In conclusion, PD-1 and PD-L1 expressions may be useful as biomarkers to predict treatment responses to NAC in breast cancer. Above all, PD-L1 expression may also be useful as biomarkers for more effective chemotherapy in TNBC.

## Additional file

**Additional file 1: Fig S1.** Overall survival analysis of the correlation with outcome. Analysis of the correlation with outcome of all 177 patients, overall survival (OS) was also significantly longer in patients with low, compared to high, PD-1 and PD-L1 expressions (p = 0.048, log-rank) (p = 0.022, log-rank) (**A**, **B**). OS did not differ significantly between patients with low vs high PD-L2 expression (p = 0.615, log-rank) (**C**). In 61 TNBC cases, OS was also significantly longer in patients with low, compared to patients with high, PD-1 expression (p = 0.021, log-rank), but OS was not significantly different based on PD-L1 expression (p = 0.155, log-rank) (**D**, **E**). DFS and OS were also not significantly different based on PD-L2 expression (p = 0.595, log-rank) (**F**). In 45 HER2^+^BC cases, OS was also not significantly longer in patients with low, compared to patients with high, PD-1, PD-L1 and PD-L2 expressions (p = 0.673, p = 0.620, p = 0.749, log-rank, respectively) (**G**–**I**).

## Abbreviations

CTLA: cytotoxic T-lymphocyte-associated protein
PD: programmed cell death
RCC: renal cell carcinoma
TNBC: triple-negative breast cancer
PD-L: programmed cell death-ligand
TILs: tumor-infiltrating lymphocytes
NAC: neoadjuvant chemotherapy
CT: computed tomography
US: ultrasonography
ER: estrogen receptor
PR: progesteron receptor
HER: human epidermal growth factor receptor
HER2^+^BC: HER2-positive breast cancer (HER2^+^BC)
RECIST: response evaluation criteria in solid tumors
pCR: pathological complete response
OS: overall survival
DFS: disease-free survival
FISH: fluorescent in situ hybridization
CEP: centromere
FOVs: fields of view
CTLs: cytotoxic T lymphocytes
ICD: immunogenic cell death
Tregs: regulatory T cells
MDSCs: myeloid-derived suppressor cells

## References

[1] Schreiber RD, Old LJ, Smyth MJ (2011). Cancer immunoediting: integrating immunity’s roles in cancer suppression and promotion. Science.

[2] Couzin-Frankel J (2013). Breakthrough of the year 2013, Cancer immunotherapy. Science.

[3] Chen L, Flies DB (2013). Molecular mechanisms of T cell co-stimulation and co-inhibition. Nat Rev Immunol.

[4] Iwai Y, Ishida M, Tanaka Y, Okazaki T, Honjo T, Minato N (2002). Involvement of PD-L1 on tumor cells in the escape from host immune system and tumor immunotherapy by PD-L1 blockade. Proc Natl Acad Sci USA.

[5] Robert C, Long GV, Brady B, Dutriaux C, Maio M, Mortier L, Hassel JC, Rutkowski P, McNeil C, Kalinka-Warzocha E (2015). Nivolumab in previously untreated melanoma without BRAF mutation. N Engl J Med.

[6] Wolchok JD, Kluger H, Callahan MK, Postow MA, Rizvi NA, Lesokhin AM, Segal NH, Ariyan CE, Gordon RA, Reed K (2013). Nivolumab plus ipilimumab in advanced melanoma. N Engl J Med.

[7] Topalian SL, Sznol M, McDermott DF, Kluger HM, Carvajal RD, Sharfman WH, Brahmer JR, Lawrence DP, Atkins MB, Powderly JD (2014). Survival, durable tumor remission, and long-term safety in patients with advanced melanoma receiving nivolumab. J Clin Oncol.

[8] Momtaz P, Postow MA (2014). Immunologic checkpoints in cancer therapy: focus on the programmed death-1 (PD-1) receptor pathway. Pharmgenom Pers Med.

[9] Rosenberg SA, Yang JC, Restifo NP (2004). Cancer immunotherapy: moving beyond current vaccines. Nat Med.

[10] Muenst S, Soysal SD, Gao F, Obermann EC, Oertli D, Gillanders WE (2013). The presence of programmed death 1 (PD-1)-positive tumor-infiltrating lymphocytes is associated with poor prognosis in human breast cancer. Breast Cancer Res Treat.

[11] Sun S, Fei X, Mao Y, Wang X, Garfield DH, Huang O, Wang J, Yuan F, Sun L, Yu Q (2014). PD-1(+) immune cell infiltration inversely correlates with survival of operable breast cancer patients. Cancer Immunol Immunother.

[12] Muenst S, Schaerli AR, Gao F, Daster S, Trella E, Droeser RA, Muraro MG, Zajac P, Zanetti R, Gillanders WE (2014). Expression of programmed death ligand 1 (PD-L1) is associated with poor prognosis in human breast cancer. Breast Cancer Res Treat.

[13] Stagg J, Allard B (2013). Immunotherapeutic approaches in triple-negative breast cancer: latest research and clinical prospects. Ther Adv Med Oncol.

[14] Zou W, Chen L (2008). Inhibitory B7-family molecules in the tumour microenvironment. Nat Rev Immunol.

[15] Hamanishi J, Mandai M, Abiko K, Matsumura N, Baba T, Yoshioka Y, Kosaka K, Konishi I (2011). The comprehensive assessment of local immune status of ovarian cancer by the clustering of multiple immune factors. Clin Immunol.

[16] Hamanishi J, Mandai M, Iwasaki M, Okazaki T, Tanaka Y, Yamaguchi K, Higuchi T, Yagi H, Takakura K, Minato N (2007). Programmed cell death 1 ligand 1 and tumor-infiltrating CD8^+^ T lymphocytes are prognostic factors of human ovarian cancer. Proc Natl Acad Sci USA.

[17] Konishi J, Yamazaki K, Azuma M, Kinoshita I, Dosaka-Akita H, Nishimura M (2004). B7-H1 expression on non-small cell lung cancer cells and its relationship with tumor-infiltrating lymphocytes and their PD-1 expression. Clin Cancer Res.

[18] Fridman WH, Pages F, Sautes-Fridman C, Galon J (2012). The immune contexture in human tumours: impact on clinical outcome. Nat Rev Cancer.

[19] Greene FL, Sobin LH (2009). A worldwide approach to the TNM staging system: collaborative efforts of the AJCC and UICC. J Surg Oncol.

[20] Goldhirsch A, Wood WC, Coates AS, Gelber RD, Thurlimann B, Senn HJ (2011). Strategies for subtypes–dealing with the diversity of breast cancer: highlights of the St. Gallen international expert consensus on the primary therapy of early breast cancer 2011. Ann Oncol.

[21] Mauri D, Pavlidis N, Ioannidis JP (2005). Neoadjuvant versus adjuvant systemic treatment in breast cancer: a meta-analysis. J Natl Cancer Inst.

[22] Mieog JS, van der Hage JA, van de Velde CJ (2007). Preoperative chemotherapy for women with operable breast cancer. Cochrane Database Syst Rev..

[23] Buzdar AU, Valero V, Ibrahim NK, Francis D, Broglio KR, Theriault RL, Pusztai L, Green MC, Singletary SE, Hunt KK (2007). Neoadjuvant therapy with paclitaxel followed by 5-fluorouracil, epirubicin, and cyclophosphamide chemotherapy and concurrent trastuzumab in human epidermal growth factor receptor 2-positive operable breast cancer: an update of the initial randomized study population and data of additional patients treated with the same regimen. Clin Cancer Res.

[24] Eisenhauer EA, Therasse P, Bogaerts J, Schwartz LH, Sargent D, Ford R, Dancey J, Arbuck S, Gwyther S, Mooney M (2009). New response evaluation criteria in solid tumours: revised RECIST guideline (version 1.1). Eur J Cancer.

[25] McShane LM, Altman DG, Sauerbrei W, Taube SE, Gion M, Clark GM (2005). Statistics subcommittee of the NCIEWGoCD: reporting recommendations for tumor marker prognostic studies (REMARK). J Natl Cancer Inst.

[26] Kashiwagi S, Yashiro M, Takashima T, Aomatsu N, Kawajiri H, Ogawa Y, Onoda N, Ishikawa T, Wakasa K, Hirakawa K (2013). c-Kit expression as a prognostic molecular marker in patients with basal-like breast cancer. Br J Surg.

[27] Umemura S, Kurosumi M, Moriya T, Oyama T, Arihiro K, Yamashita H, Umekita Y, Komoike Y, Shimizu C, Fukushima H (2006). Immunohistochemical evaluation for hormone receptors in breast cancer: a practically useful evaluation system and handling protocol. Breast Cancer.

[28] Wolff AC, Hammond ME, Hicks DG, Dowsett M, McShane LM, Allison KH, Allred DC, Bartlett JM, Bilous M, Fitzgibbons P (2013). Recommendations for human epidermal growth factor receptor 2 testing in breast cancer: American Society of Clinical Oncology/College of American Pathologists clinical practice guideline update. J Clin Oncol.

[29] Salgado R, Denkert C, Demaria S, Sirtaine N, Klauschen F, Pruneri G, Wienert S, Van den Eynden G, Baehner FL, Penault-Llorca F (2015). The evaluation of tumor-infiltrating lymphocytes (TILs) in breast cancer: recommendations by an international TILs working group 2014. Ann Oncol.

[30] Asano Y, Kashiwagi S, Goto W, Kurata K, Noda S, Takashima T, Onoda N, Tanaka S, Ohsawa M, Hirakawa K (2016). Tumour-infiltrating CD8 to FOXP3 lymphocyte ratio in predicting treatment responses to neoadjuvant chemotherapy of aggressive breast cancer. Br J Surg.

[31] Kashiwagi S, Asano Y, Goto W, Takada K, Takahashi K, Noda S, Takashima T, Onoda N, Tomita S, Ohsawa M (2017). Use of tumor-infiltrating lymphocytes (TILs) to predict the treatment response to eribulin chemotherapy in breast cancer. PLoS ONE.

[32] Nomi T, Sho M, Akahori T, Hamada K, Kubo A, Kanehiro H, Nakamura S, Enomoto K, Yagita H, Azuma M (2007). Clinical significance and therapeutic potential of the programmed death-1 ligand/programmed death-1 pathway in human pancreatic cancer. Clin Cancer Res.

[33] Mathot L, Stenninger J (2012). Behavior of seeds and soil in the mechanism of metastasis: a deeper understanding. Cancer Sci.

[34] Hanahan D, Weinberg RA (2011). Hallmarks of cancer: the next generation. Cell.

[35] Freeman GJ, Long AJ, Iwai Y, Bourque K, Chernova T, Nishimura H, Fitz LJ, Malenkovich N, Okazaki T, Byrne MC (2000). Engagement of the PD-1 immunoinhibitory receptor by a novel B7 family member leads to negative regulation of lymphocyte activation. J Exp Med.

[36] Okazaki T, Chikuma S, Iwai Y, Fagarasan S, Honjo T (2013). A rheostat for immune responses: the unique properties of PD-1 and their advantages for clinical application. Nat Immunol.

[37] Dong H, Strome SE, Salomao DR, Tamura H, Hirano F, Flies DB, Roche PC, Lu J, Zhu G, Tamada K (2002). Tumor-associated B7-H1 promotes T-cell apoptosis: a potential mechanism of immune evasion. Nat Med.

[38] Houssami N, Macaskill P, von Minckwitz G, Marinovich ML, Mamounas E (2012). Meta-analysis of the association of breast cancer subtype and pathologic complete response to neoadjuvant chemotherapy. Eur J Cancer.

[39] Cortazar P, Zhang L, Untch M, Mehta K, Costantino JP, Wolmark N, Bonnefoi H, Cameron D, Gianni L, Valagussa P (2014). Pathological complete response and long-term clinical benefit in breast cancer: the CTNeoBC pooled analysis. Lancet.

[40] Zitvogel L, Kepp O, Kroemer G (2011). Immune parameters affecting the efficacy of chemotherapeutic regimens. Nat Rev Clin Oncol.

[41] Vacchelli E, Galluzzi L, Fridman WH, Galon J, Sautes-Fridman C, Tartour E, Kroemer G (2012). Trial watch: chemotherapy with immunogenic cell death inducers. Oncoimmunology.

[42] Yaguchi T, Sumimoto H, Kudo-Saito C, Tsukamoto N, Ueda R, Iwata-Kajihara T, Nishio H, Kawamura N, Kawakami Y (2011). The mechanisms of cancer immunoescape and development of overcoming strategies. Int J Hematol.

