# Supplementary figures and images for: Prediction of treatment responses to neoadjuvant chemotherapy in triple-negative breast cancer by analysis of immune checkpoint protein expression
Source: J Transl Med. 2018 Apr 4;16:87. doi: 10.1186/s12967-018-1458-y (PMC5883348; doi:10.1186/s12967-018-1458-y)

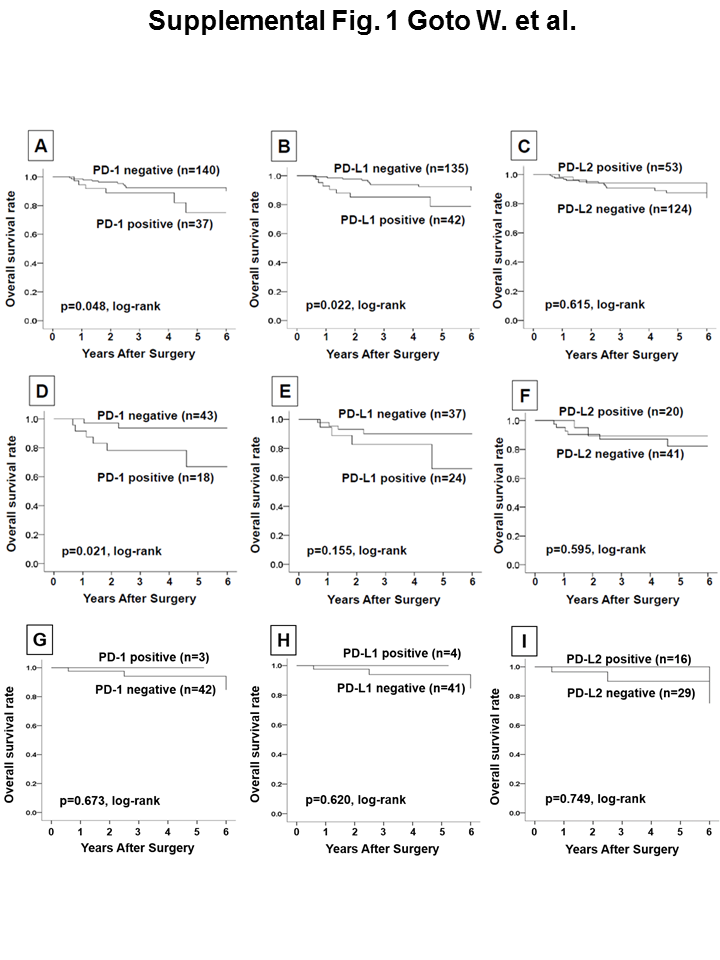

Supplement: Supplementary file 1 — Additional file 1: Fig S1. Overall survival analysis of the correlation with outcome. Analysis of the correlation with outcome of all 177 patients, overall survival (OS) was also significantly longer in patients with low, compared to high, PD-1 and PD-L1 expressions (p = 0.048, log-rank) (p = 0.022, log-rank) (A, B). OS did not differ significantly between patients with low vs high PD-L2 expression (p = 0.615, log-rank) (C). In 61 TNBC cases, OS was also significantly longer in patients with low, compared to patients with high, PD-1 expression (p = 0.021, log-rank), but OS was not significantly different based on PD-L1 expression (p = 0.155, log-rank) (D, E). DFS and OS were also not significantly different based on PD-L2 expression (p = 0.595, log-rank) (F). In 45 HER2+BC cases, OS was also not significantly longer in patients with low, compared to patients with high, PD-1, PD-L1 and PD-L2 expressions (p = 0.673, p = 0.620, p = 0.749, log-rank, respectively) (G–I). [file 12967_2018_1458_MOESM1_ESM.tif]
